# Supplementary material for: Early-adulthood spike in protein translation drives aging via juvenile hormone/germline signaling
Source: Nat Commun. 2023 Aug 18;14:5021. doi: 10.1038/s41467-023-40618-x (PMC10439225; doi:10.1038/s41467-023-40618-x)
Supplement: Supplementary file 1 — Supplementary Information [file 41467_2023_40618_MOESM1_ESM.pdf]

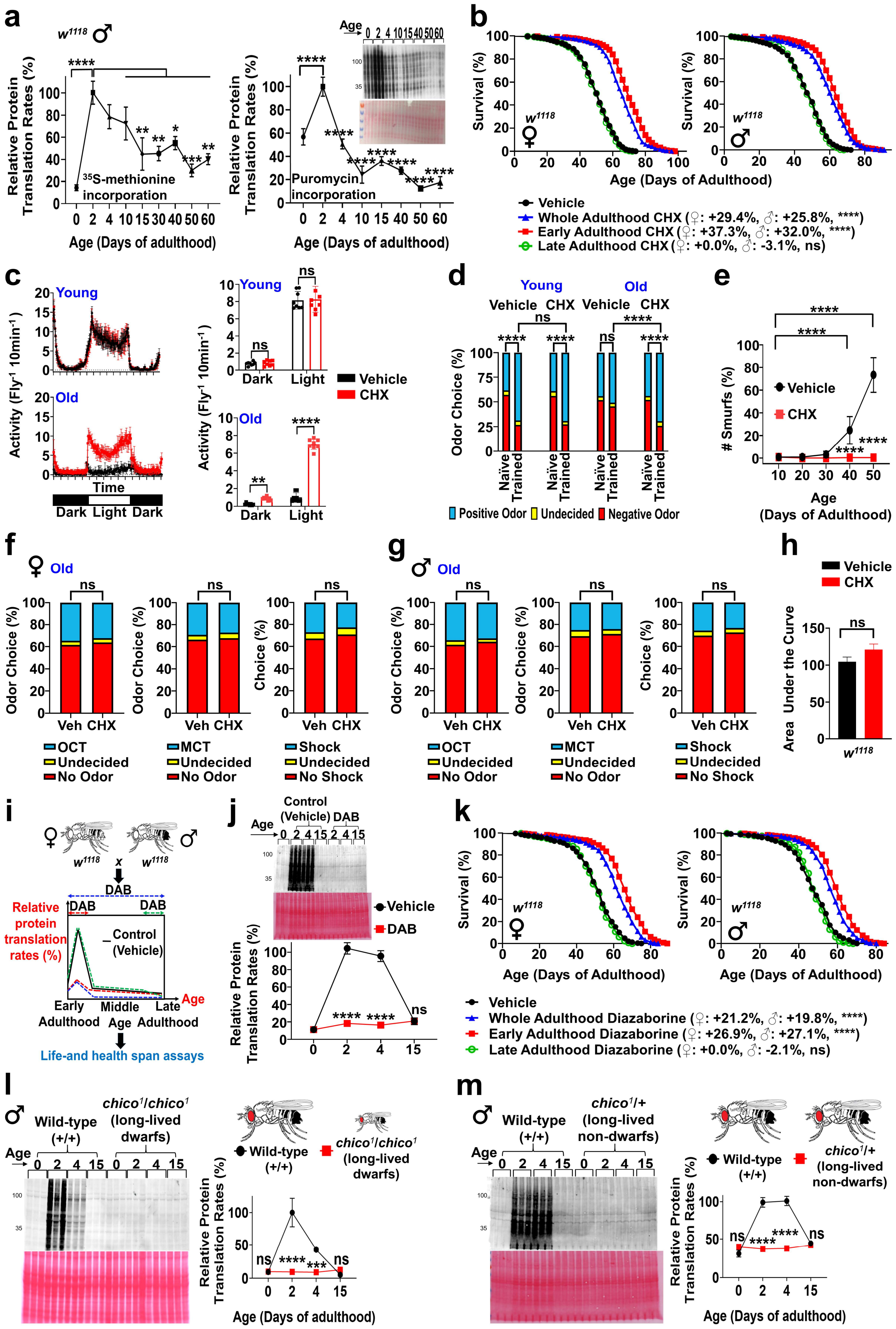

a
w1118 ♂
Relative Protein Translation Rates (%)
35S-methionine incorporation
Age (Days of adulthood)
Puromycin incorporation
Age
b
Survival (%)
Age (Days of Adulthood)
Vehicle
Whole Adulthood CHX (♀: +29.4%, ♂: +25.8%, ****)
Early Adulthood CHX (♀: +37.3%, ♂: +32.0%, ****)
Late Adulthood CHX (♀: +0.0%, ♂: -3.1%, ns)
c
Young
Old
Activity (Fly-1 10min-1)
Time
Dark Light Dark
Vehicle
CHX
d
Young Old
Vehicle CHX Vehicle CHX
Odor Choice (%)
Naïve Trained
Positive Odor Undecided Negative Odor
e
Smurfs (%)
Vehicle
CHX
Age (Days of Adulthood)
f
♀ Old
Odor Choice (%)
Choice (%)
Veh CHX
OCT Undecided No Odor
MCT Undecided No Odor
Shock Undecided No Shock
g
♂ Old
h
Vehicle
CHX
Area Under the Curve
w1118
i
Relative protein translation rates (%)
DAB
Control (Vehicle)
Early Adulthood Middle Age Late Adulthood
Age
Life-and health span assays
j
Control (Vehicle) DAB
Vehicle
DAB
k
Survival (%)
Age (Days of Adulthood)
Vehicle
Whole Adulthood Diazaborine (♀: +21.2%, ♂: +19.8%, ****)
Early Adulthood Diazaborine (♀: +26.9%, ♂: +27.1%, ****)
Late Adulthood Diazaborine (♀: +0.0%, ♂: -2.1%, ns)
l
♂ Wild-type (+/+)
chico1/chico1 (long-lived dwarfs)
Relative Protein Translation Rates (%)
Age (Days of adulthood)
m
♂ Wild-type (+/+)
chico1/+ (long-lived non-dwarfs)

Supplementary Fig. 1: Pharmacologically blocking the early-adulthood PT spike improves lifespan and healthspan. a, PT across ages in male w^{1118} flies, determined by (left) ^{35}S-methionine incorporation normalized to protein content (n=3) and (right) puromycin incorporation normalized to Ponceau staining (n=3). Analyses compare PT relative to day 2; one-way ANOVA with Dunnett's post-hoc test. **b,** Late-adulthood (day 30-40) 1µM CHX does not significantly alter lifespan of female and male w^{1118} flies. For both sexes, early-adulthood (day 0-10) CHX prolongs lifespan just like whole-adulthood CHX. Each sex: n=250/group; log-rank test. **c,** Early-adulthood (day 0-10) 1µM CHX reduces age-related deficits in spontaneous activity. Young=day 12, old=day 50. n=200♂/group; two-way ANOVA with Sidak post-hoc test. **d,** Early-adulthood (day 0-10) CHX reduces age-related cognitive deficits in olfaction aversion training. Young=day 12, old=day 50. n=200♂/group; Chi-square test. **e,** Early-adulthood (day 0-10) CHX prevents age-related deficits in gut-barrier integrity in Smurf assays. n=250♂/group; two-way ANOVA with Sidak post-hoc test. Early-adulthood (day 0-10) CHX does not significantly alter sensorimotor responses to odors (OCT/MCT) and electric shock in old **f,** female and **g,** male w^{1118} flies. OCT=3-octanol; MCT=4-methylcyclohexanol; Veh=vehicle. Old=day 50. n=200/group; Chi-square test. **h,** Area under the curve for **Fig. 1h** was calculated to determine lifetime egg production. Early-adulthood (day 0-10) CHX does not significantly alter lifetime egg production in w^{1118} flies. Two-tailed Student's t-test. **i,** Experimental scheme to transiently manipulate PT in different life stages; 100µM diazaborine given to w^{1118} flies during early-adulthood (day 0-10), late-adulthood (day 40-50), or whole-adulthood. **j,** Early-life PT in w^{1118} flies after ±100µM diazaborine (day 0-10), determined by puromycin incorporation normalized to Ponceau staining. n=3/group; two-way ANOVA with Sidak post-hoc test. **k,** In w^{1118} flies, early-adulthood (day 0-10) diazaborine prolongs lifespan just like whole-adulthood diazaborine. Late-adulthood (day 40-50) diazaborine does not alter lifespan. Each sex: n=250/group; log-rank test. Puromycin incorporation in **l,** *chico* homozygotes (♂) and **m,** *chico* heterozygotes (♂) vs. wild-types. Absence of early-life PT spike in *chico* homozygotes and *chico* heterozygotes. n=3/group; two-way ANOVA with Sidak post-hoc test. Data shown as mean±SD. *p<0.05, **p<0.01, ***p<0.001, ****p<0.0001. Source data are provided as a Source Data file. For all statistical analyses, a 2-sided p<0.05 was accepted as statistically significant. All analyses were adjusted for multiple comparisons.

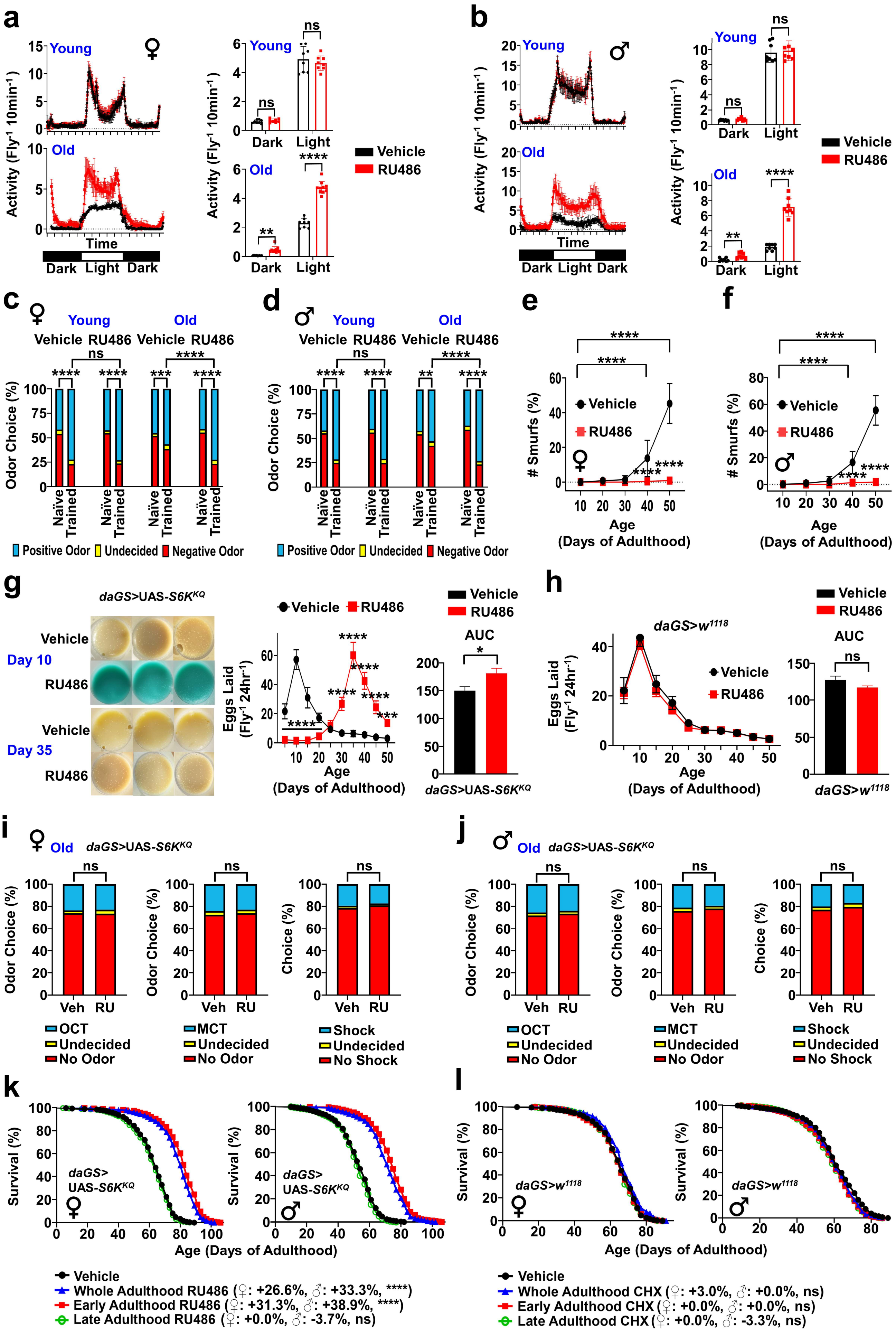

a
Young
Old
Activity (Fly-1 10min-1)
Time
Dark Light Dark
Dark
Light
ns
**
****
Vehicle
RU486
b
Young
Old
Activity (Fly-1 10min-1)
Time
Dark Light Dark
Dark
Light
ns
**
****
Vehicle
RU486
c
Young
Old
Vehicle RU486 Vehicle RU486
Odor Choice (%)
Naive
Trained
Positive Odor
Undecided
Negative Odor
d
Young
Old
Vehicle RU486 Vehicle RU486
Odor Choice (%)
Naive
Trained
Positive Odor
Undecided
Negative Odor
e
Smurfs (%)
Vehicle
RU486
Age
(Days of Adulthood)
f
Smurfs (%)
Vehicle
RU486
Age
(Days of Adulthood)
g
daGS>UAS-S6KKQ
Vehicle
Day 10
RU486
Vehicle
Day 35
RU486
Vehicle
RU486
Eggs Laid (Fly-1 24hr-1)
Age
(Days of Adulthood)
AUC
daGS>UAS-S6KKQ
h
Vehicle
RU486
daGS>w1118
Eggs Laid (Fly-1 24hr-1)
Age
(Days of Adulthood)
AUC
daGS>w1118
i
Old daGS>UAS-S6KKQ
Odor Choice (%)
Choice (%)
Veh RU
OCT
Undecided
No Odor
MCT
Undecided
No Odor
Shock
Undecided
No Shock
j
Old daGS>UAS-S6KKQ
Odor Choice (%)
Choice (%)
Veh RU
OCT
Undecided
No Odor
MCT
Undecided
No Odor
Shock
Undecided
No Shock
k
Survival (%)
daGS>
UAS-S6KKQ
Age (Days of Adulthood)
l
Survival (%)
daGS>w1118
Age (Days of Adulthood)
Vehicle
Whole Adulthood RU486 (♀: +26.6%, ♂: +33.3%, ****)
Early Adulthood RU486 (♀: +31.3%, ♂: +38.9%, ****)
Late Adulthood RU486 (♀: +0.0%, ♂: -3.7%, ns)
Vehicle
Whole Adulthood CHX (♀: +3.0%, ♂: +0.0%, ns)
Early Adulthood CHX (♀: +0.0%, ♂: +0.0%, ns)
Late Adulthood CHX (♀: +0.0%, ♂: -3.3%, ns)

Supplementary Fig. 2: Genetically blocking the early-adulthood PT spike improves lifespan and healthspan. Early-adulthood (day 0-10) 200μM RU486 reduces age-related deficits in spontaneous activity in **a,** female and **b,** male *daughterless-GeneSwitch* GAL4 (*daGS*)>UAS-$S6K^{KQ}$ flies. Young=day 12, old=day 50. n=200/group; two-way ANOVA with Sidak post-hoc test. Early-adulthood (day 0-10) 200μM RU486 reduces age-related cognitive deficits (olfaction aversion training) in **c,** female and **d,** male *daGS*>UAS-$S6K^{KQ}$ flies. Young=day 12, old=day 50. n=200/group; Chi-square test. Early-adulthood (day 0-10) 200μM RU486 prevents age-related deficits in gut-barrier integrity (Smurf assays) in **e,** female and **f,** male *daGS*>UAS-$S6K^{KQ}$ flies. n=250/group; two-way ANOVA with Sidak post-hoc test. **g,** (Left) representative images of eggs laid on vials by *daGS*>UAS-$S6K^{KQ}$ flies after 200μM RU486 treatments during early-adulthood (day 0-10). (Middle) Early-adulthood RU486 impairs egg production at young ages, delays fertility peak, and improves egg production at old ages. n=100/group; two-way ANOVA with Sidak post-hoc test. (Right) Area under the curve (AUC) was calculated to determine lifetime egg production. Early-adulthood RU486 significantly improves lifetime egg production in *daGS*>UAS-$S6K^{KQ}$ flies. Two-tailed Student's t-test. **h,** (Left) early adulthood (day 0-10) 200μM RU486 does not significantly alter egg production across ages in *daGS*>w^{1118} (control) flies. n=100/group; two-way ANOVA with Sidak post-hoc test. (Right) Area under the curve (AUC) was calculated to determine lifetime egg production. Early-adulthood RU486 does not significantly alter lifetime egg production in *daGS*>w^{1118} flies. Two-tailed Student's t-test. Early-adulthood (day 0-10) 200μM RU486 does not significantly alter sensorimotor responses to odors (OCT/MCT) and electric shock in old **i,** female and **j,** male *daGS*>UAS-$S6K^{KQ}$ flies. OCT=3-octanol; MCT=4-methylcyclohexanol; Veh=vehicle; RU=RU486. n=200/group; Chi-square test. **k,** Late-adulthood (day 30-40) 200μM RU486 does not significantly alter lifespan of female and male *daughterless-GeneSwitch* GAL4 (*daGS*)>UAS-$S6K^{KQ}$ flies. For both sexes, early-adulthood (day 0-10) RU486 prolongs lifespan just like whole-adulthood RU486. Each sex: n=250/group; log-rank test. **l,** RU486 itself does not significantly alter lifespan at any adult stages in female and male *daGS*>w^{1118} (control) flies. 200μM RU486 was given to *daGS*>w^{1118} flies during early-adulthood (day 0-10), late-adulthood (day 40-50), or whole-adulthood. Each sex: n=250/group; log-rank test. Data shown as mean±SD. *p<0.05, **p<0.01, ***p<0.001, ****p<0.0001. Source data are provided as a Source Data file. For all statistical analyses, a 2-sided p<0.05 was accepted as statistically significant. All analyses were adjusted for multiple comparisons.

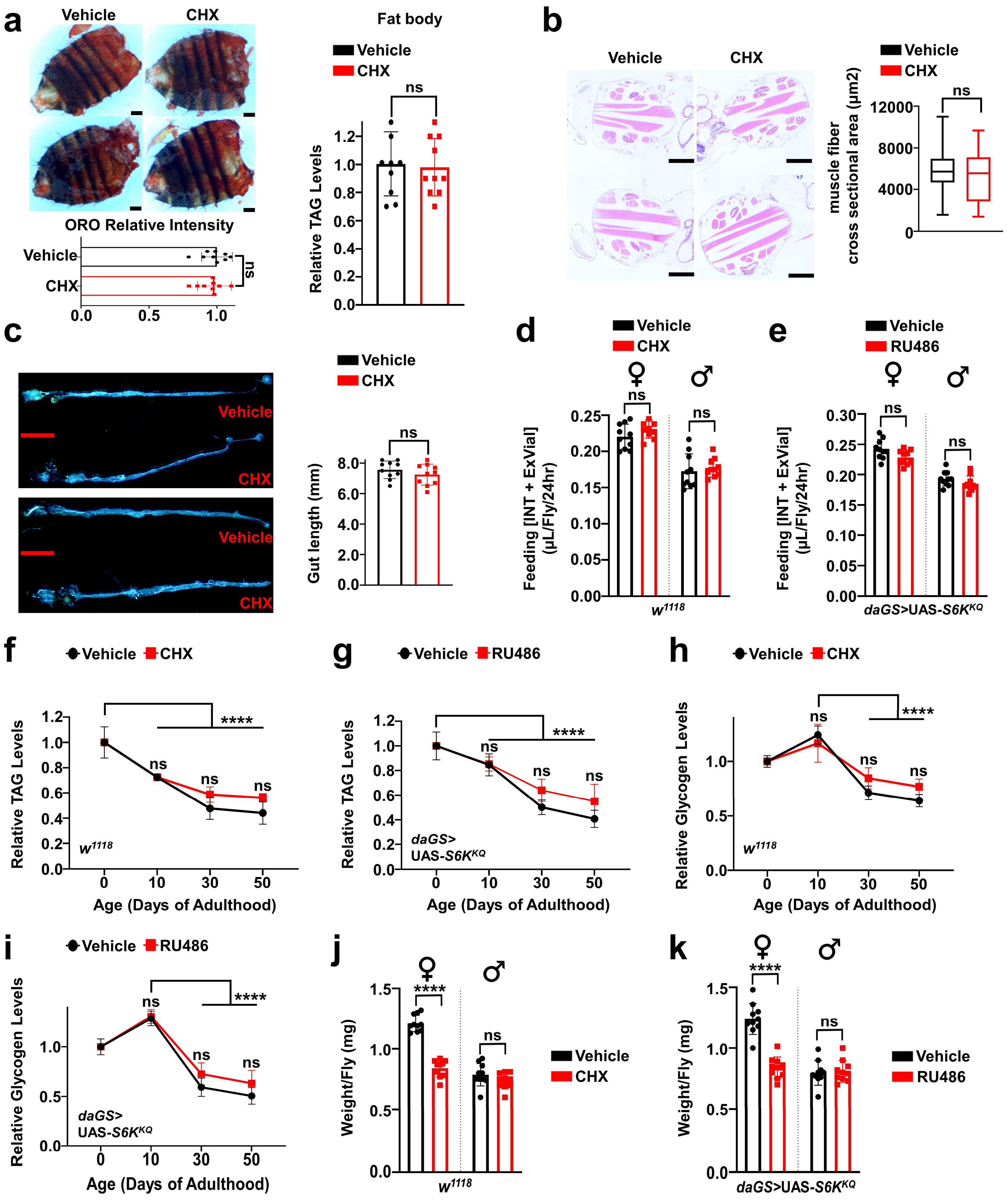

a
Vehicle
CHX
ORO Relative Intensity
Vehicle
CHX
ns
Fat body
Vehicle
CHX
ns
Relative TAG Levels
b
Vehicle
CHX
Vehicle
CHX
ns
muscle fiber cross sectional area (μm2)
c
Vehicle
CHX
Vehicle
CHX
Vehicle
CHX
ns
Gut length (mm)
d
Vehicle
CHX
ns
ns
Feeding [INT + ExVial] (μL/Fly/24hr)
w^{1118}
e
Vehicle
RU486
ns
ns
Feeding [INT + ExVial] (μL/Fly/24hr)
daGS>UAS-$S6K^{KQ}$
f
Vehicle
CHX
****
ns
Relative TAG Levels
w^{1118}
Age (Days of Adulthood)
g
Vehicle
RU486
****
ns
Relative TAG Levels
daGS> UAS-$S6K^{KQ}$
Age (Days of Adulthood)
h
Vehicle
CHX
****
ns
Relative Glycogen Levels
w^{1118}
Age (Days of Adulthood)
i
Vehicle
RU486
****
ns
Relative Glycogen Levels
daGS> UAS-$S6K^{KQ}$
Age (Days of Adulthood)
j
****
ns
Weight/Fly (mg)
w^{1118}
Vehicle
CHX
k
****
ns
Weight/Fly (mg)
daGS>UAS-$S6K^{KQ}$
Vehicle
RU486

Supplementary Fig. 3: Lifespan benefits from blocking the early-adulthood PT spike are not driven by RU486, food intake, triglyceride/glycogen levels, fat body storage, or body weight. a, Early-adulthood (day 0-10) CHX does not significantly alter Oil Red O (ORO) stain and triglyceride (TAG) levels of dissected carcasses/fat bodies. n=10/group. Two-tailed Student's t-test. **b,** Early-adulthood (day 0-10) CHX does not significantly alter the cross sectional area of muscle fibers. n=10/group; 107 fibrils for CHX group and 102 fibrils for vehicle group. Two-tailed Student's t-test. **c,** Early-adulthood (day 0-10) CHX does not significantly alter the length of gut. n=10/group. Two-tailed Student's t-test. **d,** Early-adulthood (day 0-10) CHX does not significantly alter food intake in w^{1118} flies of both sexes. Food intake was measured based on consumption/excretion of dye-containing food, as described in detail in **Methods**. INT=the dye inside the flies; ExVial=the dye excreted by flies on the wall of the vials. For each sex, N=15 flies x 10 vials/group were used. Two-tailed Student's t-test. **e,** For both sexes, early-adulthood (day 0-10) RU486 does not significantly alter food intake in *daGS*>UAS-$S6K^{KQ}$. For each sex, N=15 flies x 10 vials/group were used. Two-tailed Student's t-test. **f,** Early-adulthood (day 0-10) CHX does not significantly alter triglyceride levels in w^{1118} flies across ages. Triglyceride levels were normalized to total protein content. Triplicates of 10 female flies/age group were used. Two-way ANOVA with Sidak post-hoc test. **g,** Early-adulthood (day 0-10) RU486 does not significantly alter triglyceride levels in *daGS*>UAS-$S6K^{KQ}$ flies across ages. Triglyceride levels were normalized to total protein content. Triplicates of 10 female flies/age group were used. Two-way ANOVA with Sidak post-hoc test. **h,** Early-adulthood (day 0-10) CHX does not significantly alter glycogen levels in w^{1118} flies across ages. Glycogen levels were normalized to total protein content. Triplicates of 10 female flies/age group were used. Two-way ANOVA with Sidak post-hoc test. **i,** Early-adulthood (day 0-10) RU486 does not significantly alter glycogen levels in *daGS*>UAS-$S6K^{KQ}$ flies across ages. Glycogen levels were normalized to total protein content. Triplicates of 10 female flies/age group were used. Two-way ANOVA with Sidak post-hoc test. **j,** Early-adulthood (day 0-10) CHX decreases body weight in female but not in male w^{1118} flies. For each sex, N=10 flies x 10 vials/group were used. Two-tailed Student's t-test. **k,** Early-adulthood (day 0-10) RU486 decreases body weight in female but not in male *daGS*>UAS-$S6K^{KQ}$ flies. For each sex, N=10 flies x 10 vials/group were used. Two-tailed Student's t-test. Data shown as mean±SD. *$p<0.05$, **$p<0.01$, ***$p<0.001$, ****$p<0.0001$. Source data are provided as a Source Data file. For all statistical analyses, a 2-sided p<0.05 was accepted as statistically significant. All analyses were adjusted for multiple comparisons.

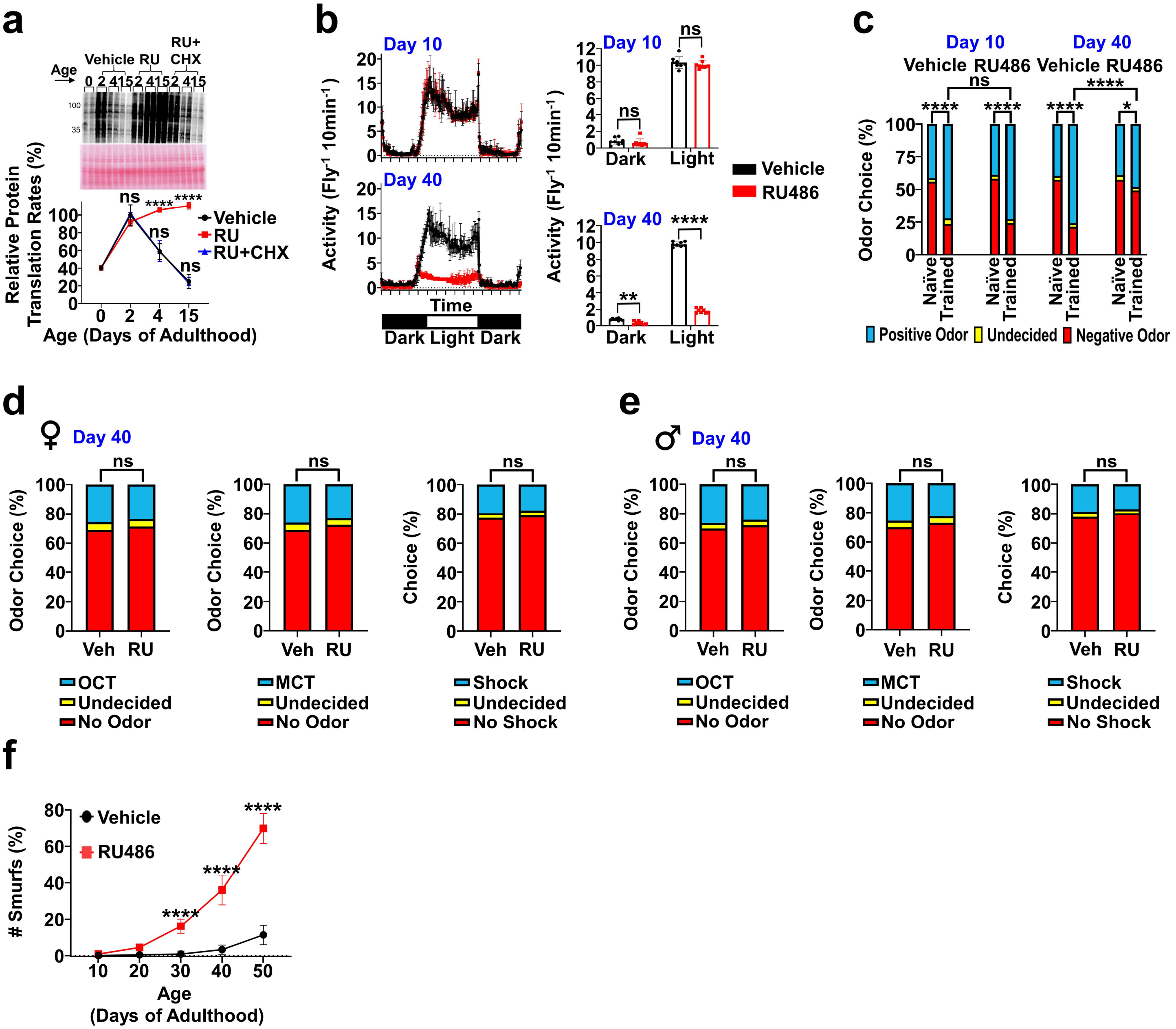

a
Age
Vehicle RU RU+ CHX
0 2 4 15 2 4 15 2 4 15
100
35
Relative Protein Translation Rates (%)
Vehicle
RU
RU+CHX
ns
****
****
ns
ns
Age (Days of Adulthood)
b
Day 10
Day 40
Activity (Fly-1 10min-1)
Time
Dark Light Dark
Dark
Light
ns
ns
**
****
Vehicle
RU486
c
Day 10
Day 40
Vehicle RU486 Vehicle RU486
Odor Choice (%)
Naïve
Trained
****
ns
****
*
Positive Odor
Undecided
Negative Odor
d
♀ Day 40
Odor Choice (%)
Choice (%)
Veh RU
ns
OCT
MCT
Shock
Undecided
No Odor
No Shock
e
♂ Day 40
f
Smurfs (%)
Vehicle
RU486
Age
(Days of Adulthood)

Supplementary Fig. 4: Preventing age-related decline in PT shortens male healthspan. 200μM RU486, 200μM RU486+1μM CHX, or vehicle was given to *daughterless-GeneSwitch* GAL4 (*daGS*)>UAS-*S6K*^{TE} flies after day 2. **a,** Puromycin incorporation in *daGS*>UAS-*S6K*^{TE} flies (+vehicle, RU486, or RU486+CHX after day 2). Puromycin incorporation was normalized to Ponceau staining. *S6K*^{TE} overexpression after day 2 prevents the age-related decline in PT, but concurrent CHX treatments re-enable flies to suppress PT with age just like controls. Two-way ANOVA with Tukey post-hoc test. n=3. **b,** *S6K*^{TE} overexpression after day 2 impairs locomotion of male flies at day 40. n=200/group; two-way ANOVA with Sidak post-hoc test. **c,** *S6K*^{TE} overexpression after day 2 impairs cognition of male flies in olfaction aversion training at day 40. n=200/group; Chi-square test. *S6K*^{TE} overexpression after day 2 does not significantly alter sensorimotor responses to odors (OCT/MCT) and electric shock in **d,** female and **e,** male flies at day 40. OCT=3-octanol; MCT=4-methylcyclohexanol; Veh=vehicle; RU=RU486. For each sex, n=200/group; Chi-square test. **f,** *S6K*^{TE} overexpression after day 2 causes premature defects in gut-barrier integrity of male flies in Smurf assays. n=250/group; two-way ANOVA with Sidak post-hoc test. Data shown as mean±SD. *p<0.05, **p<0.01, ***p<0.001, ****p<0.0001. Source data are provided as a Source Data file. For all statistical analyses, a 2-sided p<0.05 was accepted as statistically significant. All analyses were adjusted for multiple comparisons.

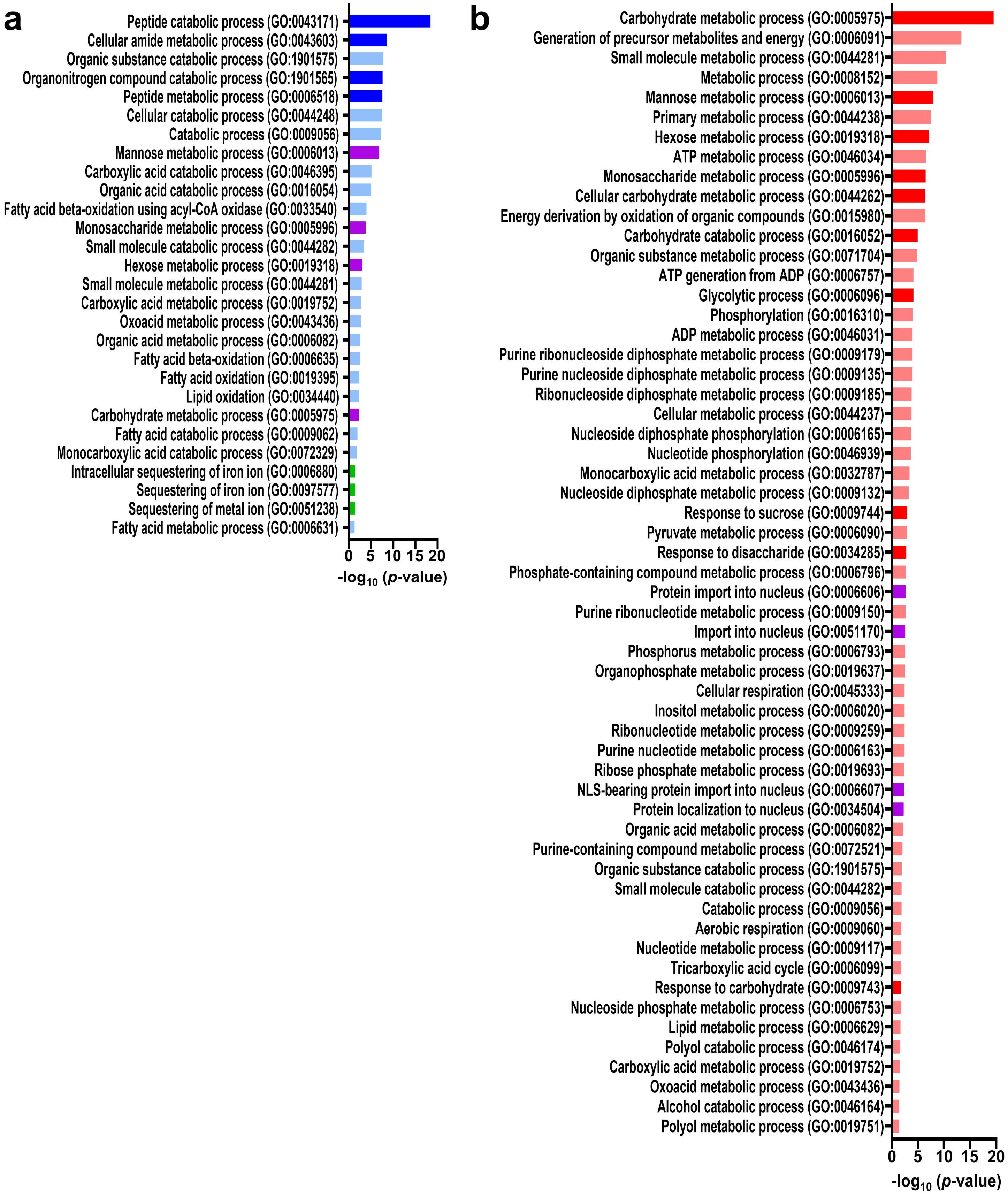

Supplementary Fig. 5: During the early-life PT rise, fat and protein catabolism are downregulated, whereas protein nuclear import and ATP/carbohydrate metabolism are upregulated. Gene Ontology (GO) analysis of proteins **a,** downregulated and **b,** upregulated during early-adulthood (from day 0 to day 2) in Triton X-100 soluble fractions, sorted by *p*-value.

a

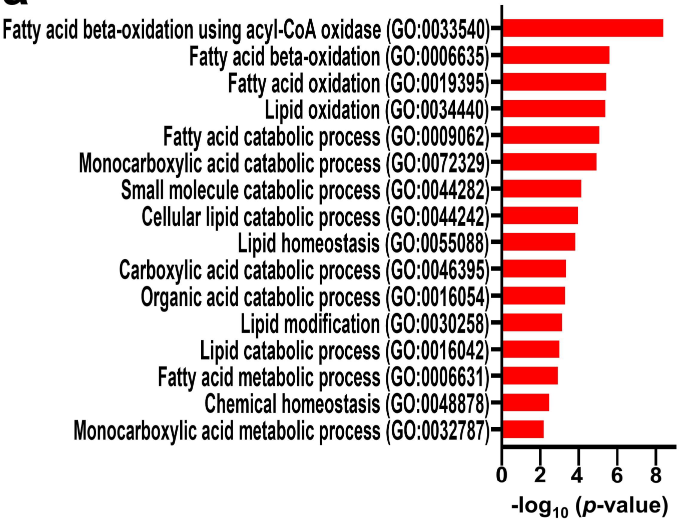

b

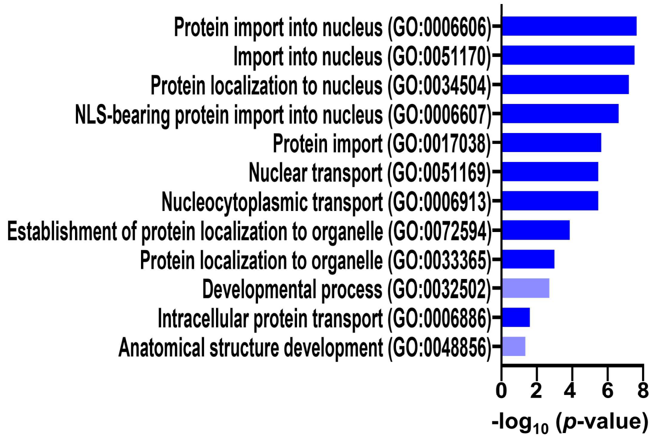

c

	β [95% CI]	p value	Inference
**Females**			
CHX	-1.896 [-2.108 to -1.686]	<0.0001	Reduces mortality
Met	0.6068 [0.4281 to 0.7856]	<0.0001	Increases mortality
CHX x Met	0.768 [0.5062 to 1.031]	<0.0001	Antagonism
**Males**			
CHX	-2.054 [-2.274 to -1.837]	<0.0001	Reduces mortality
RU486	0.4401 [0.4983 to 0.8573]	<0.0001	Increases mortality
CHX x RU486	1.172 [0.8991 to 1.447]	<0.0001	Antagonism

d

	β [95% CI]	p value	Inference
**Females**			
CA ablation	-1.605 [-1.806 to -1.405]	<0.0001	Reduces mortality
CHX	-2.181 [-2.393 to -1.970]	<0.0001	Reduces mortality
CA ablation x CHX	1.509 [1.243 to 1.775]	<0.0001	Antagonism
**Males**			
CA ablation	-1.794 [-1.997 to -1.591]	<0.0001	Reduces mortality
CHX	-2.145 [-2.353 to -1.937]	<0.0001	Reduces mortality
CA ablation x CHX	1.606 [1.339 to 1.874]	<0.0001	Antagonism

e

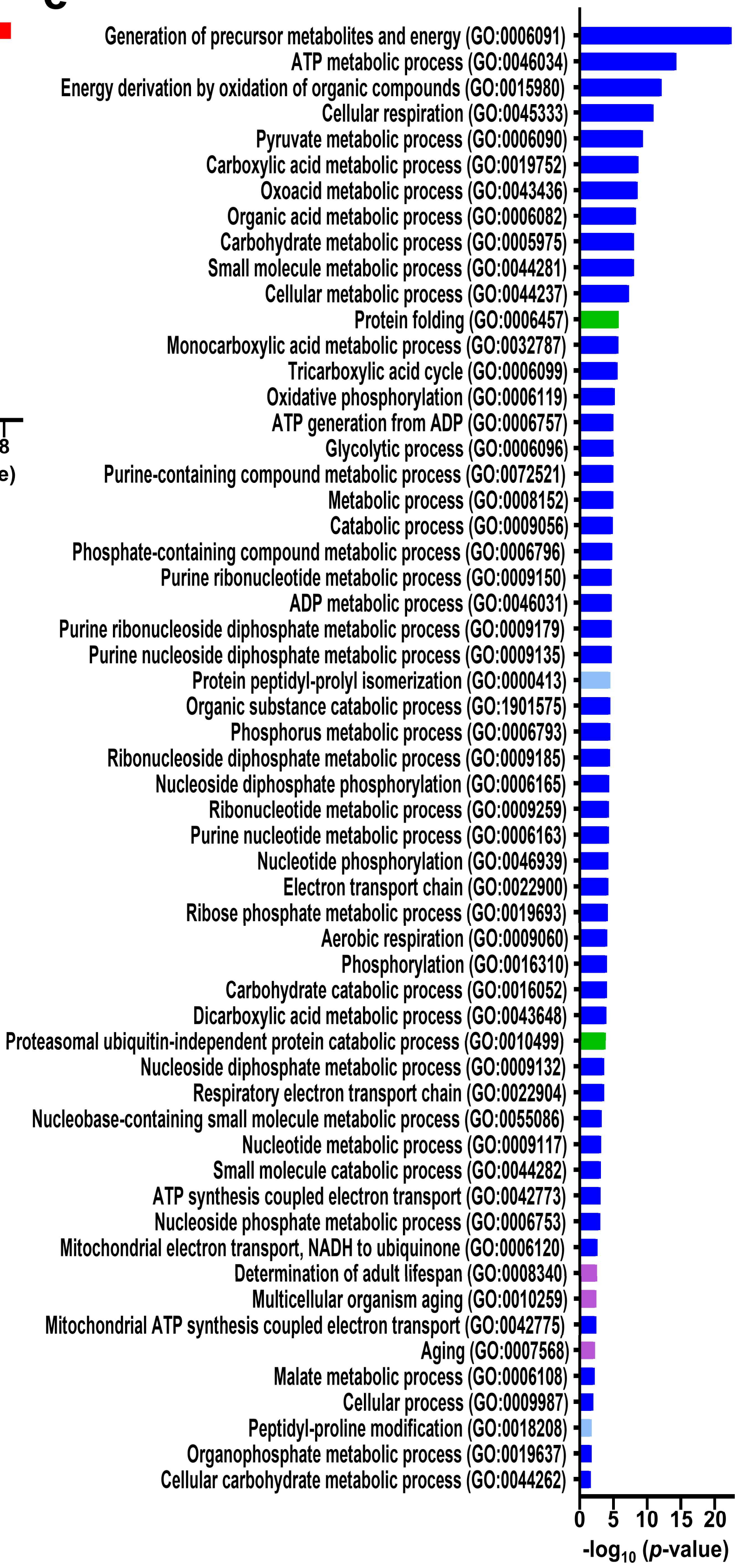

Supplementary Fig. 6: Blocking the early-life PT spike reverses lipid metabolic changes at young ages while decreasing insoluble proteins involved with ATP/carbohydrate metabolism, proteasome, and aging at old ages. Gene Ontology (GO) analysis of proteins **a,** upregulated and **b,** downregulated in Triton X-100 soluble fractions after 1µM CHX treatments during early-adulthood (day 0-2), sorted by *p*-value. Blocking the early-life PT spike upregulated fat catabolism and downregulated protein nuclear import. Proportional hazard analyses for survival curves in **c, Fig. 4f** and **d, Fig. 4g**. Coefficient β for single treatment: β significantly less than zero indicates reduction in mortality; β significantly greater than zero indicates elevated mortality. Coefficient β for interaction terms: β significantly different from zero indicates treatment interactions where the effect of two treatments differs from expectation from product of single treatment. Antagonism inferred when significant treatment interaction increases mortality (positive β) relative to expected product of two single treatments. **e,** GO analysis of proteins downregulated at old ages (day 50) in Triton X-100 insoluble fractions after 1µM CHX treatments during early-adulthood (day 0-10), sorted by p-value. Source data are provided as a Source Data file. For all statistical analyses, a 2-sided $p<0.05$ was accepted as statistically significant. All analyses were adjusted for multiple comparisons.

a

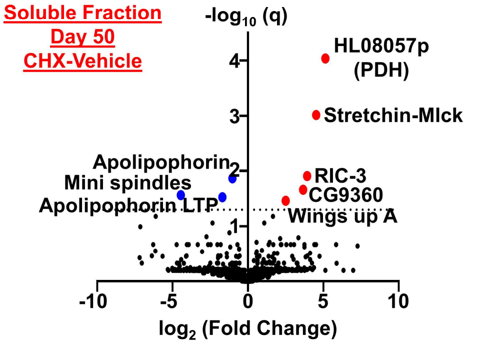

Up-regulated proteins

Proteins	q value	log_2 (Fold Change)
HL08057p (PDH, photoreceptor dehydrogenase)	0.000092	5.134099
Stretchin-Mlck (myosin light chain kinase), isoform U	0.000960	4.525470
RIC-3 (acetylcholine receptor chaperone)	0.012406	3.927233
Wings up A, isoform K	0.034659	2.505737
CG9360	0.021868	3.661777

Down-regulated proteins

Proteins	q value	log_2 (Fold Change)
Apolipophorin	0.013662	-1.02588
Mini spindles, isoform D	0.027446	-4.45198
Apolipoprotein lipid transfer particle (LTP)	0.029769	-1.69689

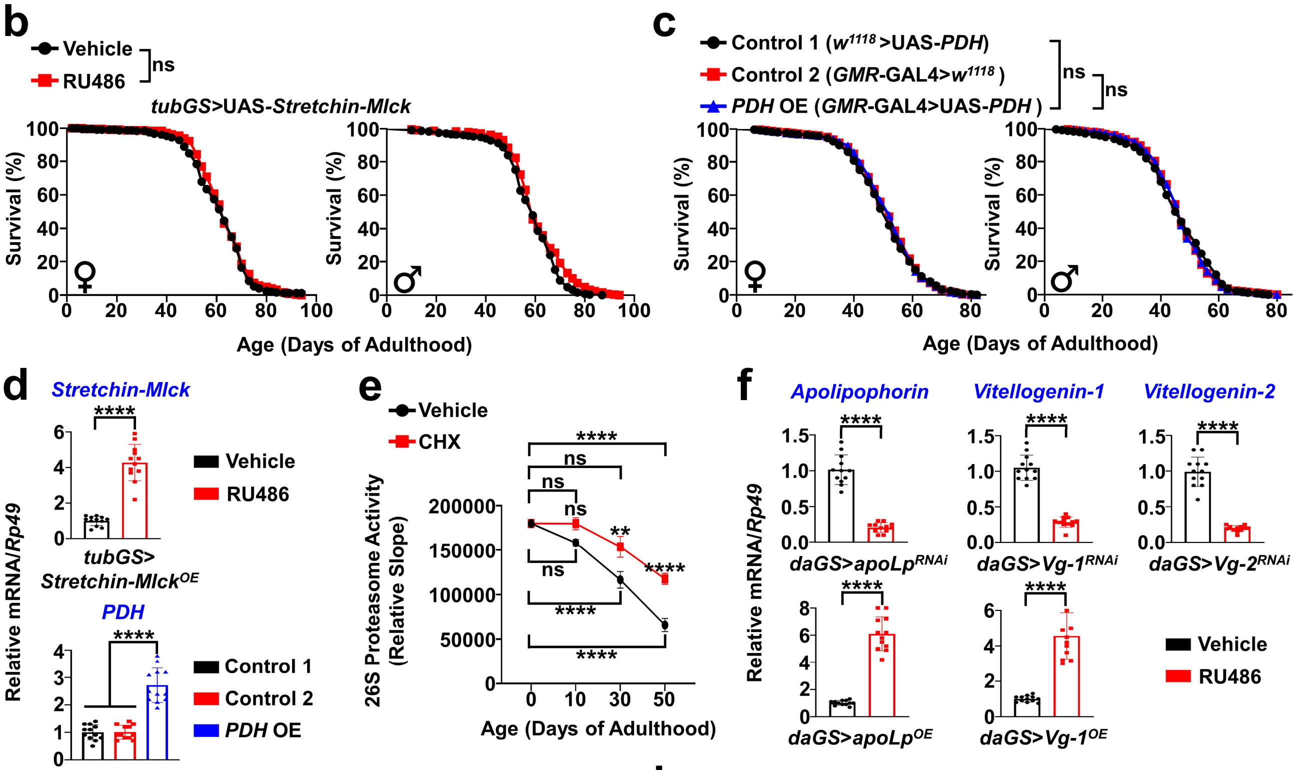

g

	β [95% CI]	p value	Inference
daGS>UAS-*apoLp* (Females)			
CHX	-2.356 [-2.589 to -2.126]	<0.0001	Reduces mortality
RU486	0.7526 [0.5718 to 0.9335]	<0.0001	Increases mortality
CHX x RU486	1.06 [0.7840 to 1.338]	<0.0001	Antagonism
daGS>UAS-*apoLp* (Males)			
CHX	-2.758 [-3.016 to -2.503]	<0.0001	Reduces mortality
RU486	0.4401 [0.5667 to 0.9255]	<0.0001	Increases mortality
CHX x RU486	0.6755 [0.9816 to 1.556]	<0.0001	Antagonism
daGS>UAS-*Vg-1* (Females)			
CHX	-1.988 [-2.206 to -1.773]	<0.0001	Reduces mortality
RU486	0.6036 [0.4242 to 0.7833]	<0.0001	Increases mortality
CHX x RU486	0.7041 [0.4389 to 0.9704]	<0.0001	Antagonism
daGS>UAS-*Vg-1* (Males)			
CHX	-1.831 [-2.037 to -1.626]	<0.0001	Reduces mortality
RU486	0.4401 [0.2636 to 0.6165]	<0.0001	Increases mortality
CHX x RU486	0.6755 [0.4198 to 0.9314]	<0.0001	Antagonism

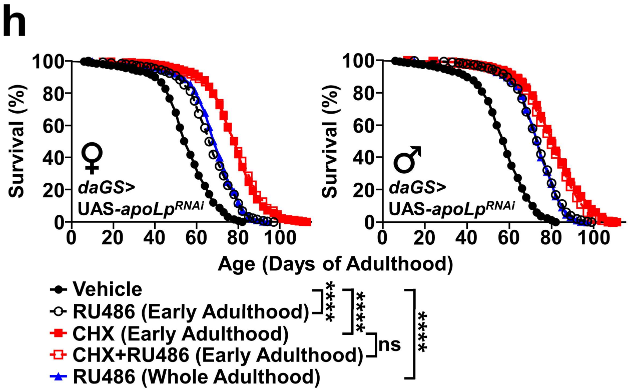

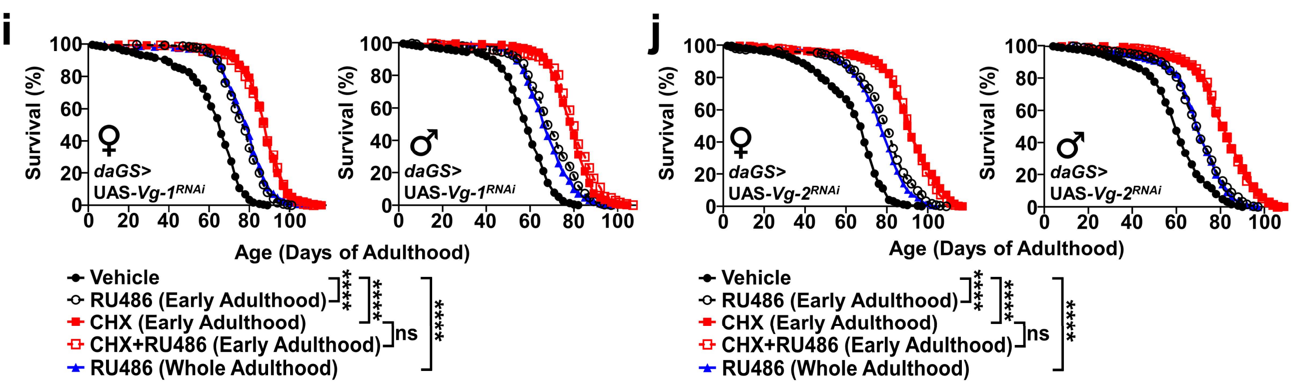

Supplementary Fig. 7: Blocking the early-life PT spike upregulates proteins important for muscle/eye functions at old ages, slows down proteasome impairment, and fails to improve lifespan upon LLTP knockdown. a, (Left) Volcano plot of proteomic analysis comparing Triton X-100 soluble fractions from day 50 flies (±1μM CHX during early-adulthood (day 0-10)). n=8/group; two-tailed Student's t-test, FDR<0.05. (Right) Tables showing up-regulated and down-regulated proteins at day 50 after ±CHX treatments during early-adulthood. **b,** Life-long overexpression of *Stretchin-Mlck* does not significantly alter lifespan in either female or male flies. For each sex, n=250/group; log-rank test. **c,** Life-long overexpression of *PDH* (photoreceptor dehydrogenase) does not significantly alter lifespan in either female or male flies. For each sex, n=250/group; log-rank test. **d,** Validation of *Stretchin-Mlck* and *PDH* overexpressing lines. (Top) qRT-PCR from whole bodies (n=12/group) indicates that mRNA levels of *Stretchin-Mlck* are elevated in *tubGS*>UAS-*Stretchin Mlck* lines treated with 200μM RU486 (day 0-10), compared with vehicle-treated uninduced controls. Two-tailed Student's t-test. (Bottom) qRT-PCR from heads (n=12/group) indicates that mRNA levels of *PDH* are elevated in *GMR*-GAL4>UAS-*PDH*, compared with w^{1118}>UAS-*PDH* (Control 1) and *GMR*-GAL4>w^{1118} (Control 2). Expressions of target genes were normalized to *Rp49*. One-way ANOVA with Tukey post-hoc test. **e,** Early adulthood (day 0-10) CHX significantly slows down the age-related decline in 26S proteasome activity in w^{1118} flies. n=12/group; Two-way ANOVA with Tukey post-hoc test. **f,** Validation of apolipophorin (*apoLp*), vitellogenin-1 (*Vg-1*), and vitellogenin-2 (*Vg-2*) RNAi lines and *apoLp* and *Vg-1* overexpressing lines. (Top) qRT-PCR from whole bodies (n=12/group) indicates that mRNA levels of *apoLp*, *Vg-1*, and *Vg-2* are decreased in RNAi lines upon RU486 treatments (day 0-10). (Bottom) qRT-PCR from whole bodies (n=12/group) indicates that mRNA levels of *apoLp* and *Vg-1* are increased in overexpression lines upon RU486 treatments (day 0-10). Two-tailed Student's t-test. **g,** Proportional hazard analyses of survival curves in **Fig. 5b** and **Fig 5d**. Coefficient β for single treatment: β significantly less than zero indicates reduction in mortality; β significantly greater than zero indicates elevated mortality. Coefficient β for interaction terms: β significantly different from zero indicates treatment interactions where the effect of two treatments differs from expectation from product of single treatment. Antagonism inferred when significant treatment interaction increases mortality (positive β) relative to expected product of two single treatments. **h,** For both sexes, knocking down *Vg-1* significantly prolongs lifespan in control flies but not in flies treated with CHX during early adulthood (day 0-10). For each sex, n=250/group; log-rank test. **i,** For both sexes, knocking down *Vg-2* significantly prolongs lifespan in control flies but not in flies treated with CHX during early adulthood (day 0-10). For each sex, n=250/group; log-rank test. **j,** For both sexes, knocking down *apoLp* significantly prolongs lifespan in control flies but not in flies treated with CHX during early adulthood (day 0-10). For each sex, n=250/group; log-rank test. Data shown as mean±SD. **p*<0.05, ***p*<0.01, ****p*<0.001, *****p*<0.0001. Source data are provided as a Source Data file. For all statistical analyses, a 2-sided p<0.05 was accepted as statistically significant. All analyses were adjusted for multiple comparisons.

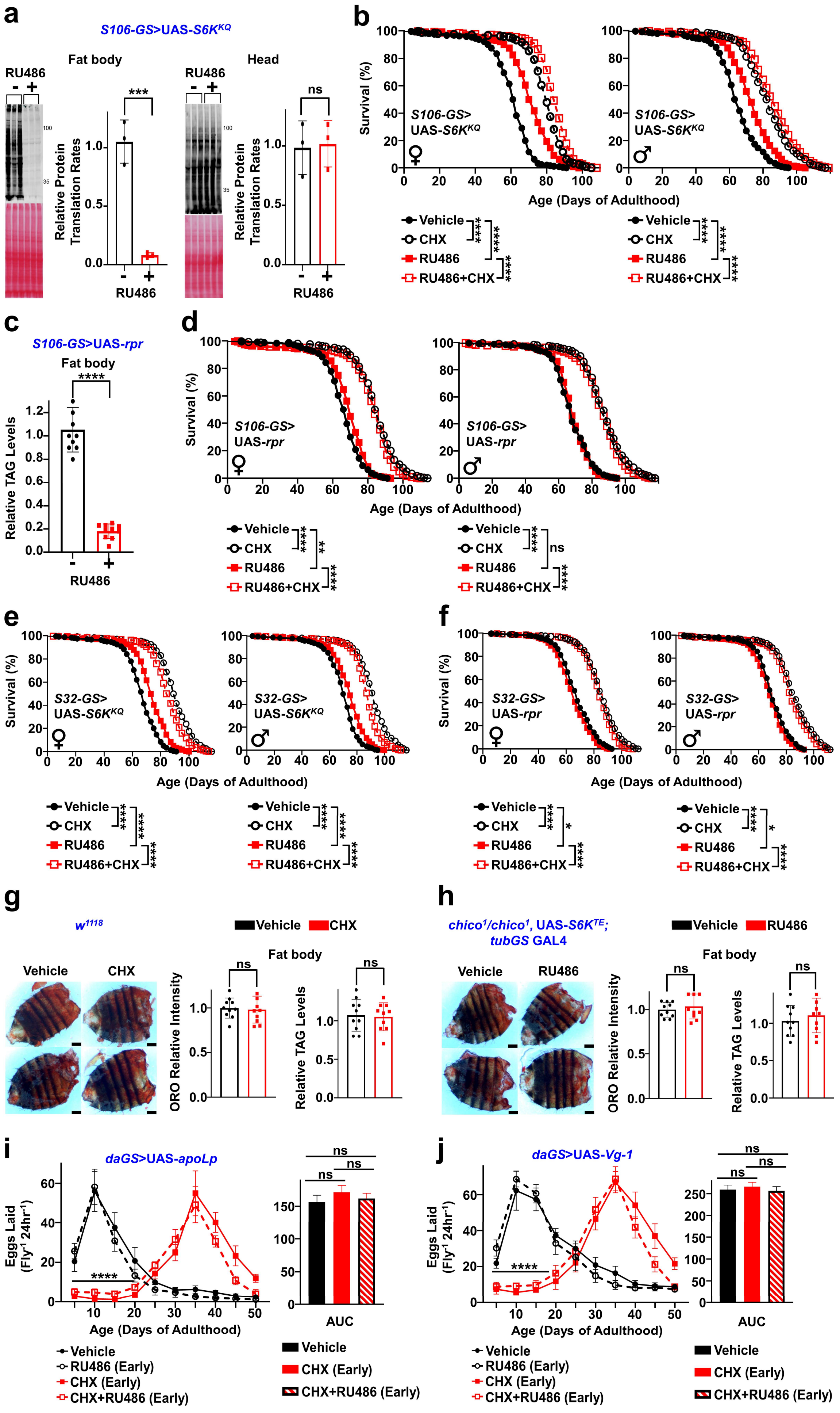
a
S106-GS>UAS-S6K^KQ
Fat body
Head
RU486
Relative Protein Translation Rates
***
ns
b
S106-GS> UAS-S6K^KQ
Survival (%)
Age (Days of Adulthood)
Vehicle
CHX
RU486
RU486+CHX
c
S106-GS>UAS-rpr
Fat body
Relative TAG Levels
****
d
S106-GS> UAS-rpr
e
S32-GS> UAS-S6K^KQ
f
S32-GS> UAS-rpr
g
w^1118
Vehicle
CHX
ORO Relative Intensity
Relative TAG Levels
h
chico^1/chico^1, UAS-S6K^TE; tubGS GAL4
RU486
i
daGS>UAS-apoLp
Eggs Laid (Fly^-1 24hr^-1)
AUC
Vehicle
RU486 (Early)
CHX (Early)
CHX+RU486 (Early)
j
daGS>UAS-Vg-1

Supplementary Fig. 8: Fat body remodeling alone is not sufficient for longevity benefits from blocking the early-life PT spike a, Puromycin incorporation in (left) abdominal fat body and (right) head of *S106-GeneSwitch* GAL4 (*S106*-GS)>UAS-$S6K^{KQ}$ flies ±200μM RU486 (day 0-day 10). Puromycin incorporation normalized to Ponceau staining. n=3/group; two-tailed Student's t-test. **b,** For both sexes, early-life CHX (1μM, day 0-day 10) improves lifespan of *S106*-GS>UAS-$S6K^{KQ}$ flies under both vehicle (control) and RU486 conditions. For each sex, n=250/group; log-rank test. **c,** Overexpressing *rpr* under the *S106* fat body driver significantly decreased levels of triglycerides in the fat body. n=10/group; two-tailed Student's t-test. **d,** For both sexes, early-life CHX (1μM, day 0-day 10) improves lifespan of *S106*-GS>UAS-*rpr* flies under both vehicle (control) and RU486 conditions. For each sex, n=250/group; log-rank test. **e,** For both sexes, early-life CHX (1μM, day 0-day 10) improves lifespan of *S32-GeneSwitch* GAL4 (*S32*-GS)>UAS-$S6K^{KQ}$ flies under both vehicle (control) and RU486 conditions. For each sex, n=250/group; log-rank test. **f,** For both sexes, early-life CHX (1μM, day 0-day 10) improves lifespan of *S32*-GS>UAS-*rpr* flies under both vehicle (control) and RU486 conditions. For each sex, n=250/group; log-rank test. **g,** Early-life CHX (1μM, day 0-day 10) does not significantly alter Oil Red O (ORO) stain and triglyceride (TAG) levels of the dissected carcasses/fat bodies in w^{1118} flies. n=10/group. Two-tailed Student's t-test. **h,** Inducing early-life PT spike with 200μM RU486 (day 0-day 4) in *chico* homozygotes does not significantly alter Oil Red O (ORO) stain and triglyceride (TAG) levels of the dissected carcasses/fat bodies. n=10/group. Two-tailed Student's t-test. **i,** Early-life CHX (1μM, day 0-day 10) impairs egg productions during early-adulthood. With simultaneous overexpression of *apoLP* (day 0-day 10), early-adulthood egg production was still significantly impaired. n=100/group; two-way ANOVA with Sidak post-hoc test. **j,** Early-life CHX (1μM, day 0-day 10) impairs egg productions during early-adulthood. With simultaneous overexpression of *Vg-1* (day 0-day 10), early-adulthood egg production was still significantly impaired. n=100/group; two-way ANOVA with Sidak post-hoc test. Data shown as mean±SD. *p<0.05, **p<0.01, ***p<0.001, ****p<0.0001. Source data are provided as a Source Data file.

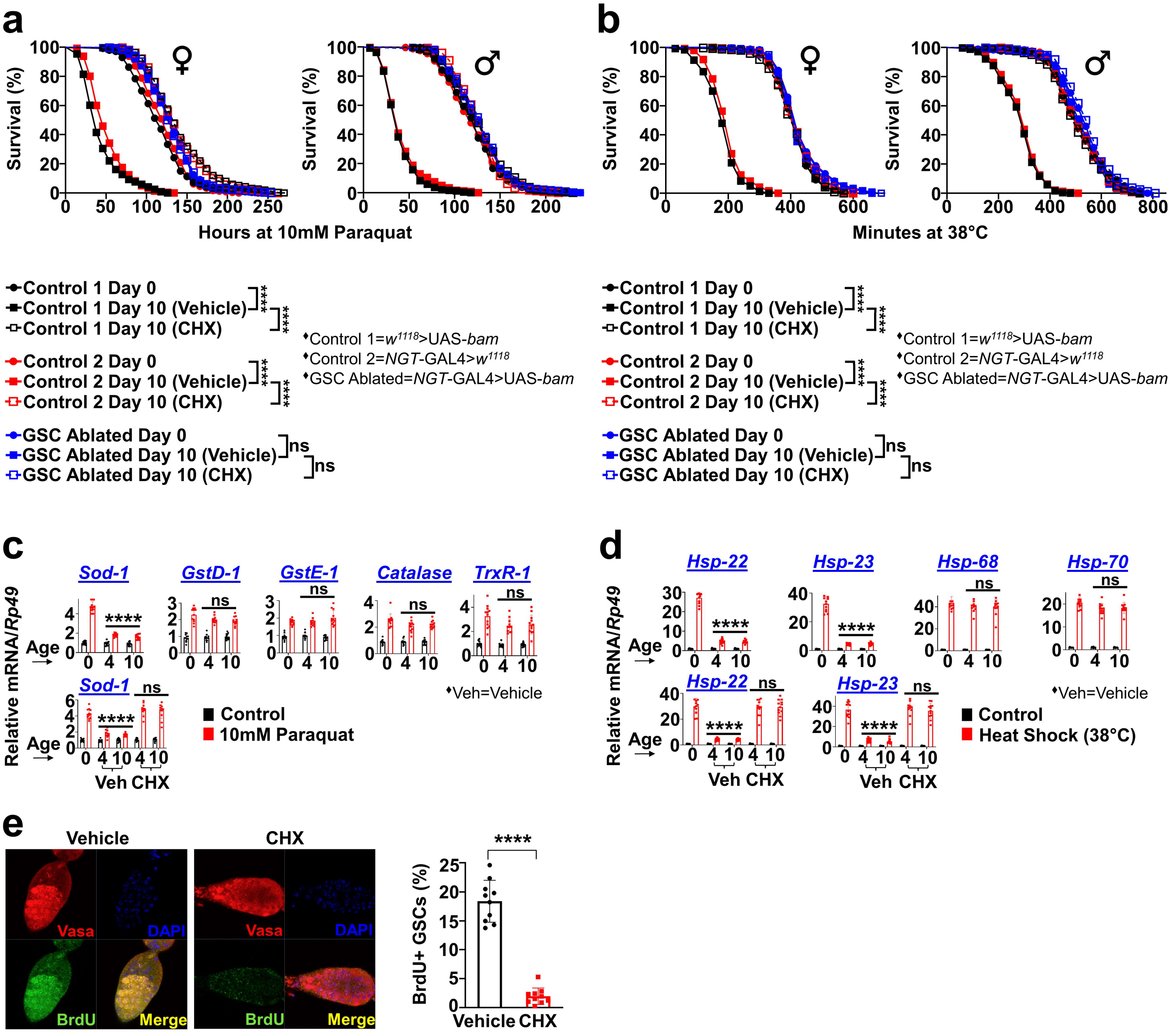

Supplementary Fig. 9: Eliminating the early-life PT spike prevents the sharp decline in stress resistance during early-adulthood via germline stem cell signaling. a, For both sexes, early adulthood (day 0-10) 1μM CHX treatments enhance oxidative stress resistance in germline stem cell (GSC)-intact flies but not in GSC-ablated flies. From day 0 to day 10, oxidative stress resistance sharply declines in GSC-intact flies but not in GSC-ablated flies and flies treated with CHX during early-adulthood. Oxidative stress:10mM paraquat; heat stress: 38°C. For each sex, n=250/group; log-rank test. **b,** For both sexes, early-adulthood (day 0-10) CHX treatments enhance heat stress resistance in GSC-intact flies but not in GSC-ablated flies. From day 0 to day 10, heat stress resistance sharply declines in GSC-intact flies but not in GSC-ablated flies and flies treated with CHX during early-adulthood. For each sex, n=250/group; log-rank test. Within 4 days, **c,** *Sod-1* inductions in response to paraquat and **d,** *Hsp-22*/*Hsp-23* inductions in response to 38°C were impaired. Inductions of other cellular stress resistance genes were not significantly affected during early-adulthood. Early adulthood CHX restores *Sod-1* inductions in response to paraquat and *Hsp-22*/*Hsp-23* inductions in response to 38°C. n=12/group; Two-way ANOVA with Tukey post-hoc test. **e**, GSCs are stained for GSC-specific antigen Vasa, BrdU, and DAPI after ±1μM CHX (day 0-5). Early-adulthood CHX reduces proliferating GSCs. n=10/group; two-tailed Student's t-test. Data shown as

mean±SD. *$p<0.05$, **$p<0.01$, ***$p<0.001$, ****$p<0.0001$. Source data are provided as a Source Data file. For all statistical analyses, a 2-sided $p<0.05$ was accepted as statistically significant. All analyses were adjusted for multiple comparisons.

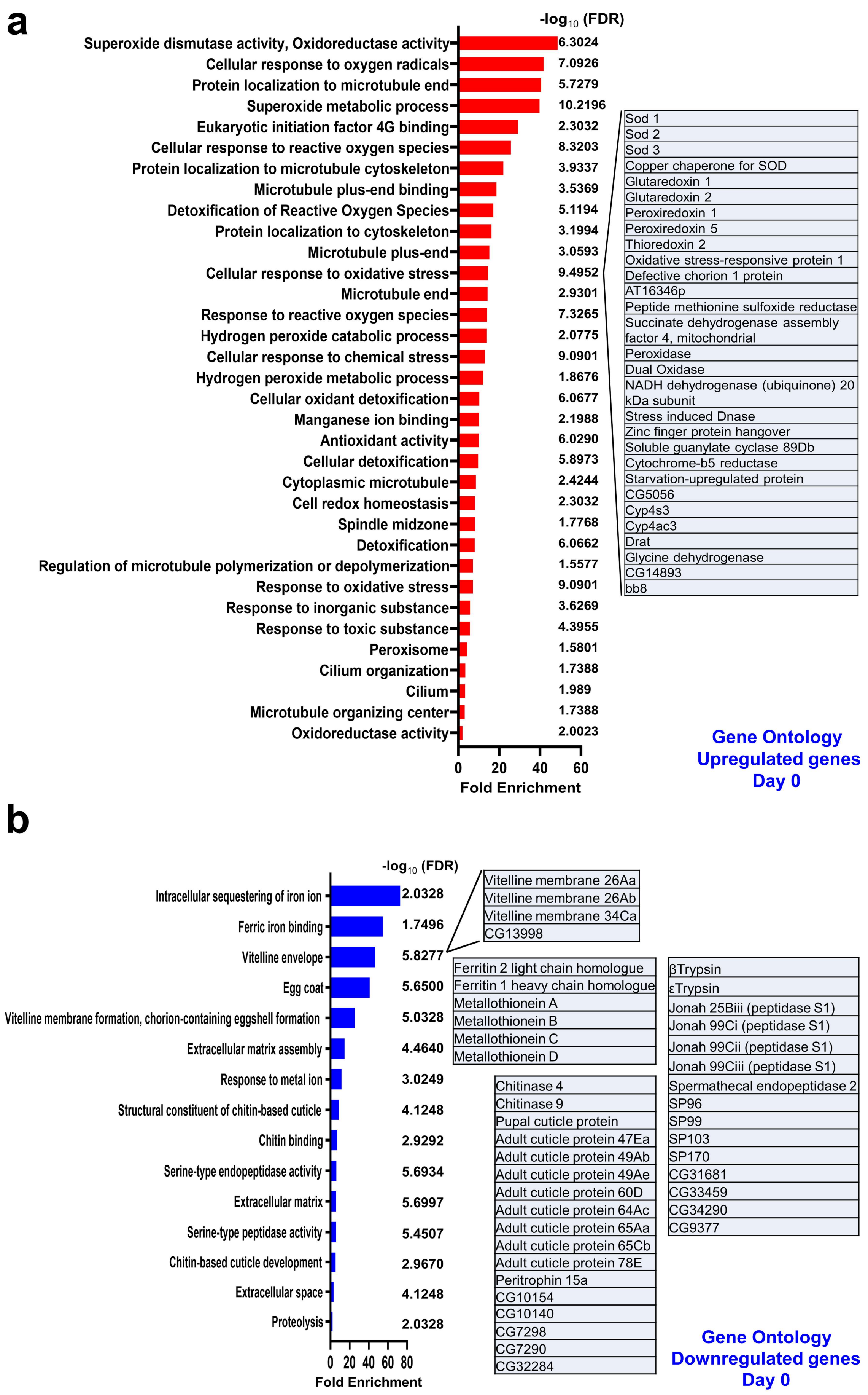

Supplementary Fig. 10: Gene ontology (GO) biological processes enriched in genes that are up-/down-regulated after paraquat in day 0 flies. 10mM paraquat for 12 hours.

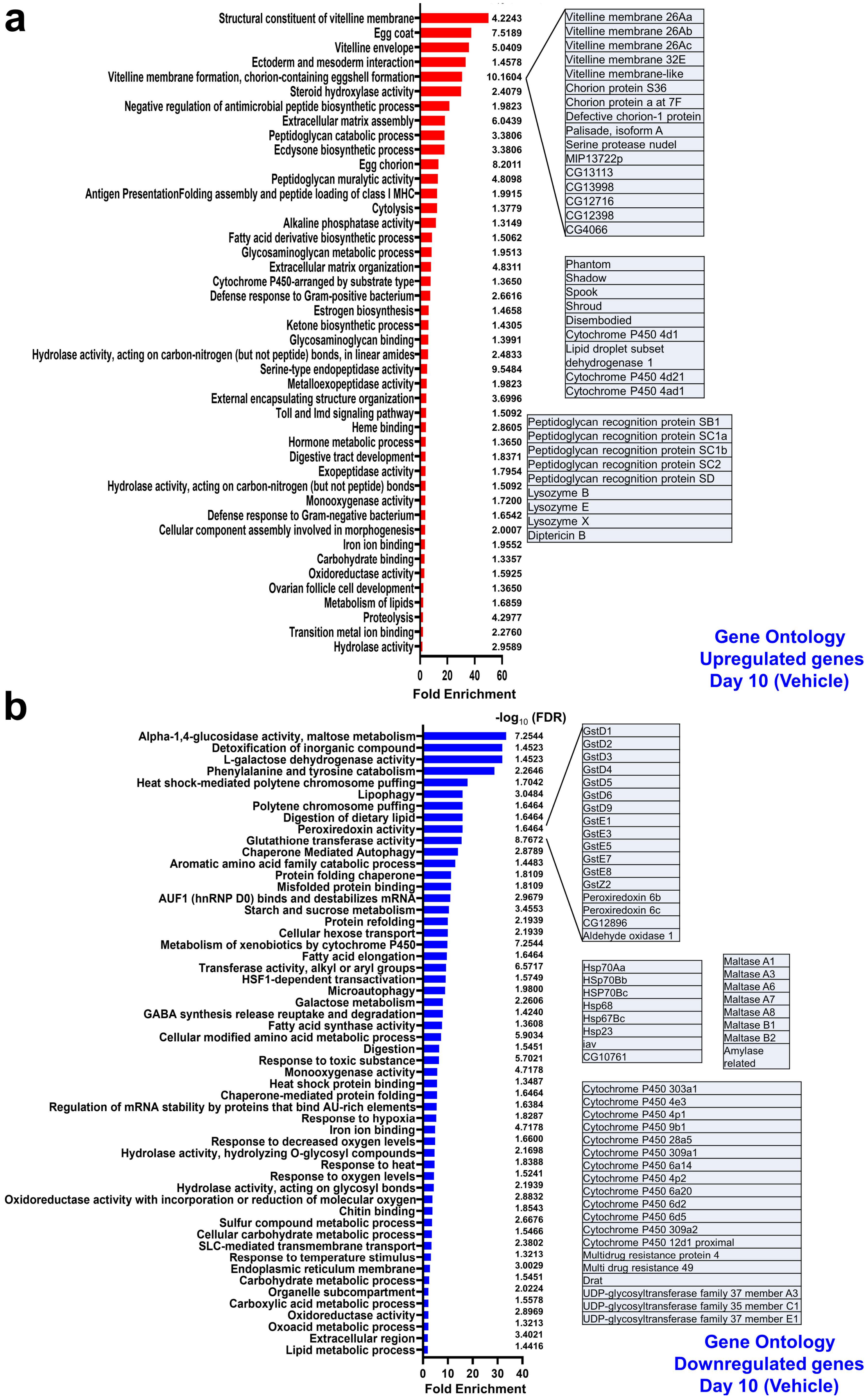

Supplementary Fig. 11: GO biological processes enriched in genes that are up-/down-regulated after paraquat in day 10 flies (vehicle). 10mM paraquat for 12 hours. Vehicle (water, day 0-day10).

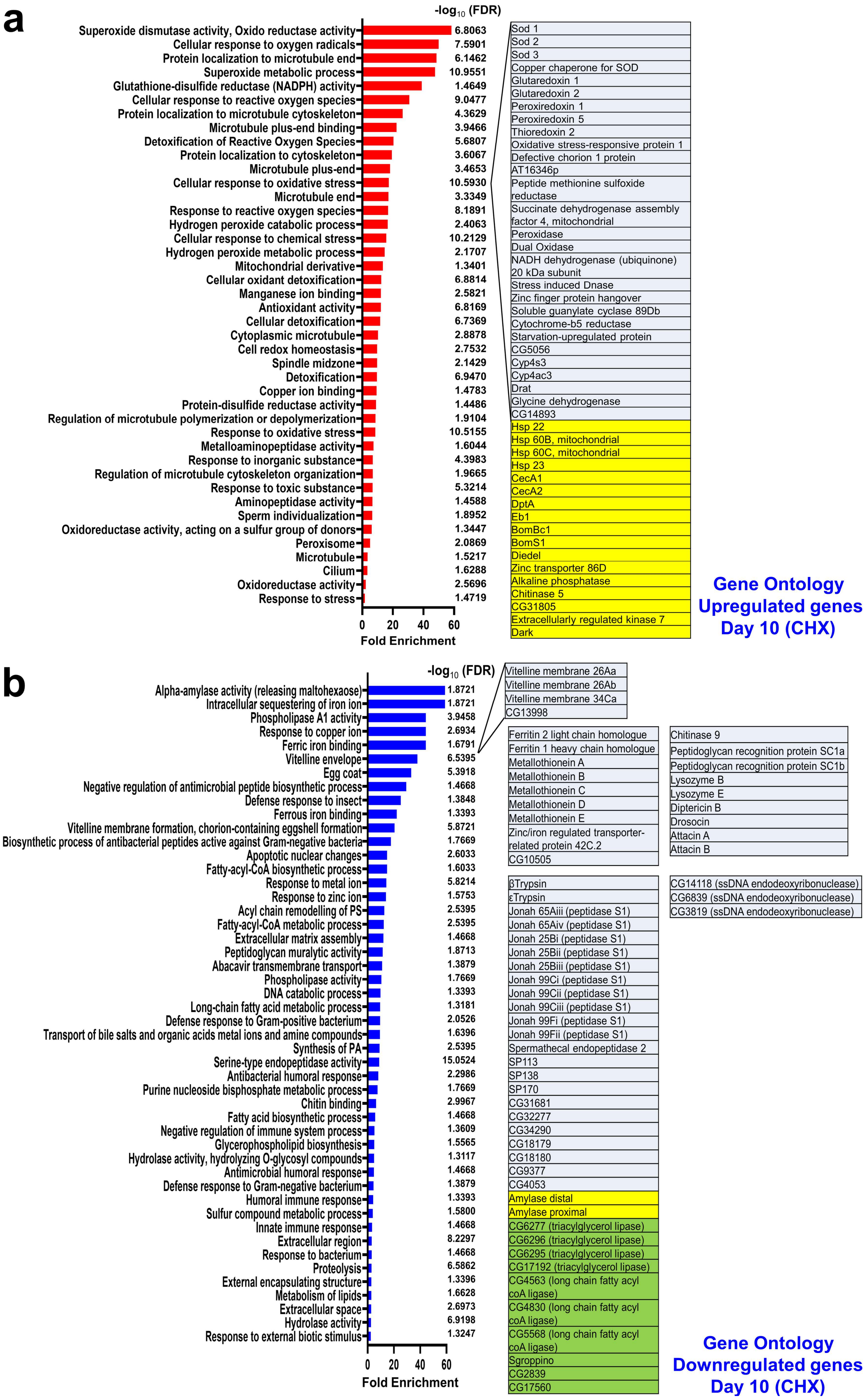
a
-log10 (FDR)
Superoxide dismutase activity, Oxido reductase activity 6.8063
Cellular response to oxygen radicals 7.5901
Protein localization to microtubule end 6.1462
Superoxide metabolic process 10.9551
Glutathione-disulfide reductase (NADPH) activity 1.4649
Cellular response to reactive oxygen species 9.0477
Protein localization to microtubule cytoskeleton 4.3629
Microtubule plus-end binding 3.9466
Detoxification of Reactive Oxygen Species 5.6807
Protein localization to cytoskeleton 3.6067
Microtubule plus-end 3.4653
Cellular response to oxidative stress 10.5930
Microtubule end 3.3349
Response to reactive oxygen species 8.1891
Hydrogen peroxide catabolic process 2.4063
Cellular response to chemical stress 10.2129
Hydrogen peroxide metabolic process 2.1707
Mitochondrial derivative 1.3401
Cellular oxidant detoxification 6.8814
Manganese ion binding 2.5821
Antioxidant activity 6.8169
Cellular detoxification 6.7369
Cytoplasmic microtubule 2.8878
Cell redox homeostasis 2.7532
Spindle midzone 2.1429
Detoxification 6.9470
Copper ion binding 1.4783
Protein-disulfide reductase activity 1.4486
Regulation of microtubule polymerization or depolymerization 1.9104
Response to oxidative stress 10.5155
Metalloaminopeptidase activity 1.6044
Response to inorganic substance 4.3983
Regulation of microtubule cytoskeleton organization 1.9665
Response to toxic substance 5.3214
Aminopeptidase activity 1.4588
Sperm individualization 1.8952
Oxidoreductase activity, acting on a sulfur group of donors 1.3447
Peroxisome 2.0869
Microtubule 1.5217
Cilium 1.6288
Oxidoreductase activity 2.5696
Response to stress 1.4719
0 20 40 60
Fold Enrichment
Sod 1
Sod 2
Sod 3
Copper chaperone for SOD
Glutaredoxin 1
Glutaredoxin 2
Peroxiredoxin 1
Peroxiredoxin 5
Thioredoxin 2
Oxidative stress-responsive protein 1
Defective chorion 1 protein
AT16346p
Peptide methionine sulfoxide reductase
Succinate dehydrogenase assembly factor 4, mitochondrial
Peroxidase
Dual Oxidase
NADH dehydrogenase (ubiquinone) 20 kDa subunit
Stress induced Dnase
Zinc finger protein hangover
Soluble guanylate cyclase 89Db
Cytochrome-b5 reductase
Starvation-upregulated protein
CG5056
Cyp4s3
Cyp4ac3
Drat
Glycine dehydrogenase
CG14893
Hsp 22
Hsp 60B, mitochondrial
Hsp 60C, mitochondrial
Hsp 23
CecA1
CecA2
DptA
Eb1
BomBc1
BomS1
Diedel
Zinc transporter 86D
Alkaline phosphatase
Chitinase 5
CG31805
Extracellularly regulated kinase 7
Dark
Gene Ontology
Upregulated genes
Day 10 (CHX)
b
-log10 (FDR)
Alpha-amylase activity (releasing maltohexaose) 1.8721
Intracellular sequestering of iron ion 1.8721
Phospholipase A1 activity 3.9458
Response to copper ion 2.6934
Ferric iron binding 1.6791
Vitelline envelope 6.5395
Egg coat 5.3918
Negative regulation of antimicrobial peptide biosynthetic process 1.4668
Defense response to insect 1.3848
Ferrous iron binding 1.3393
Vitelline membrane formation, chorion-containing eggshell formation 5.8721
Biosynthetic process of antibacterial peptides active against Gram-negative bacteria 1.7669
Apoptotic nuclear changes 2.6033
Fatty-acyl-CoA biosynthetic process 1.6033
Response to metal ion 5.8214
Response to zinc ion 1.5753
Acyl chain remodelling of PS 2.5395
Fatty-acyl-CoA metabolic process 2.5395
Extracellular matrix assembly 1.4668
Peptidoglycan muralytic activity 1.8713
Abacavir transmembrane transport 1.3879
Phospholipase activity 1.7669
DNA catabolic process 1.3393
Long-chain fatty acid metabolic process 1.3181
Defense response to Gram-positive bacterium 2.0526
Transport of bile salts and organic acids metal ions and amine compounds 1.6396
Synthesis of PA 2.5395
Serine-type endopeptidase activity 15.0524
Antibacterial humoral response 2.2986
Purine nucleoside bisphosphate metabolic process 1.7669
Chitin binding 2.9967
Fatty acid biosynthetic process 1.4668
Negative regulation of immune system process 1.3609
Glycerophospholipid biosynthesis 1.5565
Hydrolase activity, hydrolyzing O-glycosyl compounds 1.3117
Antimicrobial humoral response 1.4668
Defense response to Gram-negative bacterium 1.3879
Humoral immune response 1.3393
Sulfur compound metabolic process 1.5800
Innate immune response 1.4668
Extracellular region 8.2297
Response to bacterium 1.4668
Proteolysis 6.5862
External encapsulating structure 1.3396
Metabolism of lipids 1.6628
Extracellular space 2.6973
Hydrolase activity 6.9198
Response to external biotic stimulus 1.3247
0 20 40 60
Fold Enrichment
Vitelline membrane 26Aa
Vitelline membrane 26Ab
Vitelline membrane 34Ca
CG13998
Ferritin 2 light chain homologue
Ferritin 1 heavy chain homologue
Metallothionein A
Metallothionein B
Metallothionein C
Metallothionein D
Metallothionein E
Zinc/iron regulated transporter-related protein 42C.2
CG10505
Chitinase 9
Peptidoglycan recognition protein SC1a
Peptidoglycan recognition protein SC1b
Lysozyme B
Lysozyme E
Diptericin B
Drosocin
Attacin A
Attacin B
βTrypsin
εTrypsin
Jonah 65Aiii (peptidase S1)
Jonah 65Aiv (peptidase S1)
Jonah 25Bi (peptidase S1)
Jonah 25Bii (peptidase S1)
Jonah 25Biii (peptidase S1)
Jonah 99Ci (peptidase S1)
Jonah 99Cii (peptidase S1)
Jonah 99Ciii (peptidase S1)
Jonah 99Fi (peptidase S1)
Jonah 99Fii (peptidase S1)
Spermathecal endopeptidase 2
SP113
SP138
SP170
CG31681
CG32277
CG34290
CG18179
CG18180
CG9377
CG4053
Amylase distal
Amylase proximal
CG6277 (triacylglycerol lipase)
CG6296 (triacylglycerol lipase)
CG6295 (triacylglycerol lipase)
CG17192 (triacylglycerol lipase)
CG4563 (long chain fatty acyl coA ligase)
CG4830 (long chain fatty acyl coA ligase)
CG5568 (long chain fatty acyl coA ligase)
Sgroppino
CG2839
CG17560
CG14118 (ssDNA endodeoxyribonuclease)
CG6839 (ssDNA endodeoxyribonuclease)
CG3819 (ssDNA endodeoxyribonuclease)
Gene Ontology
Downregulated genes
Day 10 (CHX)

Supplementary Fig. 12: GO biological processes enriched in genes that are up-/down-regulated after paraquat in day 10 flies (CHX). 10mM paraquat for 12 hours. CHX (1μM, day 0-day10).
