# Supplementary material for: Early-adulthood spike in protein translation drives aging via juvenile hormone/germline signaling
Source: Nat Commun. 2023 Aug 18;14:5021. doi: 10.1038/s41467-023-40618-x (PMC10439225; doi:10.1038/s41467-023-40618-x)
Supplement: Supplementary file 3 — Description of Additional Supplementary Files [file 41467_2023_40618_MOESM3_ESM.pdf]

<u>Description of Additional Supplementary Files</u>

Supplementary Data 1: Proteomic analyses. **a.** List of all the proteins significantly upregulated during early-adulthood (from day 0 to day 2) in Triton X-100 soluble fractions. Proteins highlighted in yellow were previously shown to be important for reproduction and/or gametogenesis. **b.** List of all the proteins significantly downregulated during early-adulthood (from day 0 to day 2) in Triton X-100 soluble fractions. **c.** List of all the proteins significantly upregulated and downregulated in Triton X-100 soluble fractions after cyclohexamide (CHX, 1µM) treatments during early-adulthood (from day 0 to day 2). Proteins highlighted in yellow were previously shown to be important for reproduction and/or gametogenesis. **d.** List of all the proteins significantly downregulated at old ages (day 50) in Triton X-100 insoluble fractions after cyclohexamide (CHX, 1µM) treatments during early-adulthood (from day 0 to day 10). Significance determined by p-values corrected for false discovery rates (FDR) and multiple comparisons.
