# Supplementary material for: Early-adulthood spike in protein translation drives aging via juvenile hormone/germline signaling
Source: Nat Commun. 2023 Aug 18;14:5021. doi: 10.1038/s41467-023-40618-x (PMC10439225; doi:10.1038/s41467-023-40618-x)
Supplement: Supplementary file 5 — Reporting Summary [file 41467_2023_40618_MOESM5_ESM.pdf]

nature portfolio

Corresponding author(s): Andrew M. Pickering

Last updated by author(s): May 5, 2023

Reporting Summary

Nature Portfolio wishes to improve the reproducibility of the work that we publish. This form provides structure for consistency and transparency in reporting. For further information on Nature Portfolio policies, see our Editorial Policies and the Editorial Policy Checklist.

Statistics

For all statistical analyses, confirm that the following items are present in the figure legend, table legend, main text, or Methods section.

n/a	Confirmed	
☐	☒	The exact sample size (*n*) for each experimental group/condition, given as a discrete number and unit of measurement
☐	☒	A statement on whether measurements were taken from distinct samples or whether the same sample was measured repeatedly
☐	☒	The statistical test(s) used AND whether they are one- or two-sided<br>*Only common tests should be described solely by name; describe more complex techniques in the Methods section.*
☐	☒	A description of all covariates tested
☐	☒	A description of any assumptions or corrections, such as tests of normality and adjustment for multiple comparisons
☐	☒	A full description of the statistical parameters including central tendency (e.g. means) or other basic estimates (e.g. regression coefficient) AND variation (e.g. standard deviation) or associated estimates of uncertainty (e.g. confidence intervals)
☐	☒	For null hypothesis testing, the test statistic (e.g. *F*, *t*, *r*) with confidence intervals, effect sizes, degrees of freedom and *P* value noted<br>*Give P values as exact values whenever suitable.*
☒	☐	For Bayesian analysis, information on the choice of priors and Markov chain Monte Carlo settings
☒	☐	For hierarchical and complex designs, identification of the appropriate level for tests and full reporting of outcomes
☐	☒	Estimates of effect sizes (e.g. Cohen's *d*, Pearson's *r*), indicating how they were calculated

Our web collection on statistics for biologists contains articles on many of the points above.

Software and code

Policy information about availability of computer code

Data collection	For lifespan data collection: dLife (https://www.flidea.tech/projects)<br>For immunostaining imaging: Carl Zeiss LSM 700 confocal microscope<br>For immunoblot imaging: Odyssey® DLx Infrared Imaging System<br>For colorimetric assays :SpectraMax iD3 Multi-Mode Microplate Reader<br>For RT-qPCR: Roche LightCycler480
Data analysis	GraphPad Prism 8.0, Microsoft Office 2016 Excel, ImageJ, Image Studio 5.2 (LI-COR Biosciences), Scaffold 5.0 (Proteome Software), SoftMax Pro (Molecular Devices), Uniprot, Gene Ontologies, Zeiss Zen

For manuscripts utilizing custom algorithms or software that are central to the research but not yet described in published literature, software must be made available to editors and reviewers. We strongly encourage code deposition in a community repository (e.g. GitHub). See the Nature Portfolio guidelines for submitting code & software for further information.

Data

Policy information about availability of data

All manuscripts must include a data availability statement. This statement should provide the following information, where applicable:

- Accession codes, unique identifiers, or web links for publicly available datasets
- A description of any restrictions on data availability
- For clinical datasets or third party data, please ensure that the statement adheres to our policy

Proteomics data have been deposited at Mendeley (https://doi.org/10.17632/3k9h543hmr.1) and are publicly available as of the date of publication. All other data supporting the findings of this study are available within the article and Supplementary Information files and from the corresponding author upon request.

Field-specific reporting

Please select the one below that is the best fit for your research. If you are not sure, read the appropriate sections before making your selection.

☒ Life sciences ☐ Behavioural & social sciences ☐ Ecological, evolutionary & environmental sciences

For a reference copy of the document with all sections, see nature.com/documents/nr-reporting-summary-flat.pdf

Life sciences study design

All studies must disclose on these points even when the disclosure is negative.

Sample size	Sample size was determined to be adequate based on prior publications and the magnitude and consistency of measurable differences between groups in preliminary experiments. In many experiments, numerous flies were available and large sample sizes were used. No statistical calculations were performed to predetermine the sample size.
Data exclusions	No data were excluded from the analyses.
Replication	All of the studies were done at least 3 times in different fly cohorts and stocks (with at least 3 biological replicates) to verify the reproducibility of experimental findings. For in vitro experiments, biological replicates as well as technical replicates were used to ensure reproducibility.
Randomization	Animals were randomly allocated to vehicle or drug treatments.
Blinding	Investigators who did experiments were blinded during collections/experimental processing of samples. The sample was coded by the investigator who was not doing experiments. The code was concealed until all the experiments were done. For lifespan assays, fly vials were randomized in terms of tray position and semi-blinded to reduce impacts of environment/investigator bias.

Reporting for specific materials, systems and methods

We require information from authors about some types of materials, experimental systems and methods used in many studies. Here, indicate whether each material, system or method listed is relevant to your study. If you are not sure if a list item applies to your research, read the appropriate section before selecting a response.

Materials & experimental systems

n/a	Involved in the study	
☐	☒	Antibodies
☒	☐	Eukaryotic cell lines
☒	☐	Palaeontology and archaeology
☐	☒	Animals and other organisms
☒	☐	Human research participants
☒	☐	Clinical data
☒	☐	Dual use research of concern

Methods

n/a	Involved in the study	
☒	☐	ChIP-seq
☒	☐	Flow cytometry
☒	☐	MRI-based neuroimaging

Antibodies

Antibodies used	For Western Blot: Rabbit anti-β-actin (D6A8; 1:1000, Cell Signaling, Cat# 8457), Rabbit anti-ubiquitin (P37; 1:1000, Cell Signaling, Cat# 58395), Goat anti-mouse IgG (IRDye 800CW; 1:5000, LI-COR Biosciences, Cat# 926-32210), Goat anti-rabbit IgG (IRDye 680RD; 1:5000, LI-COR Biosciences, Cat# 926-68071), Mouse anti-puromycin (3RH11; 1:1000, Kerafast, Cat# EQ0001)<br><br>For Staining: Mouse anti-BrdU (G3G4; 1:50, Developmental Studies Hybridoma Bank), Rat-anti Vasa (1:50, Lasko and Ashburner, 1990), Alexa Fluor 488-conjugated goat anti-mouse IgG (Thermo Scientific, 1:300, Cat# A-11001), Alexa Fluor 568-conjugated goat anti-rat IgG (Thermo Scientific, 1:300, Cat# A-11077)
Validation	The specificity and applicability of all the antibodies used in our biological assays have been extensively characterized in previous publications/other laboratories and/or documentation provided by the manufacturer. Certificates of analysis are provided on supplier website.<br><br>Rabbit anti-β-actin (D6A8; 1:1000, Cell Signaling, Cat# 8457), Drosophila, validated on manufacturer's website: https://www.cellsignal.com/products/primary-antibodies/b-actin-d6a8-rabbit-mab/8457<br><br>Rabbit anti-ubiquitin (P37; 1:1000, Cell Signaling, Cat# 58395), Drosophila, validated on manufacturer's website: https://www.cellsignal.com/products/primary-antibodies/ubiquitin-p37-antibody/58395?site-search-type=Products&N=4294956287&Ntt=58395&fromPage=plp&_requestid=4587224<br><br>Mouse anti-puromycin (3RH11; 1:1000, Kerafast, Cat# EQ0001), Drosophila, validated on the prior publication: https://

www.sciencedirect.com/science/article/pii/S1550413121004174

Mouse anti-BrdU (G3G4; 1:50, Developmental Studies Hybridoma Bank), Drosophila, validated on the prior publication: https://onlinelibrary.wiley.com/doi/10.1111/j.1474-9726.2008.00379.x

Rat-anti Vasa (1:50, Lasko and Ashburner, 1990), Drosophila, validated on the prior publication: http://genesdev.cshlp.org/content/4/6/905.long

Animals and other organisms

Policy information about studies involving animals; ARRIVE guidelines recommended for reporting animal research

Laboratory animals	Following Drosophila strains were used in this study: w1118, Daughterless-GeneSwitch-GAL4 (daGS), Tubulin-GeneSwitch-GAL4 (tubGS), chico1/chico1, UAS-S6KKQ, UAS-S6KTE, Elav GeneSwitch-GAL4>UAS-hAPP; UAS-hBACE1, park13/park13, UAS-bam, UAS-apoLp, UAS-apoLp RNAi, UAS-Stretchin-Mlck, ovoD1, Aug21-GAL4, UAS-NiPp1, UAS-Vg-1 RNAi, UAS-Vg-1, UAS-Vg-2 RNAi, NGT-GAL4, GMR-GAL4, UAS-PDH, and DGRP library panel. ovoD1 and UAS/GAL4 lines were backcrossed to w1118 for 8-10 times. Transgene expression was confirmed by qRT-PCR. Both sexes of animals were used throughout experiments. Flies at various ages from young to old ages (ranging from day 0 to day 50) were used to investigate age-related changes.
Wild animals	No wild animals were used in the study.
Field-collected samples	No field collected samples were used in the study.
Ethics oversight	No ethical approval or guidance was required for the use of fruit flies/Drosophila.

Note that full information on the approval of the study protocol must also be provided in the manuscript.
